# Incidence of Dependency-Related Skin Injury in Critical Care: A Longitudinal Study

**DOI:** 10.3390/nursrep16070255

**Published:** 2026-07-21

**Authors:** Paula Balaguer Escutia, Evelin Balaguer López, Pablo Garcia Molina

**Affiliations:** 1 Intensive Care Unit, Hospital Clínico Universitario de Valencia, Blasco Ibañez w/n, 46010 Valencia, Spain; paubaes97@gmail.com; 2 Associated Research Group in Care, Blasco Ibañez w/n, 46010 Valencia, Spain; pagarmo3@uv.es; 3 Pediatric Service, Hospital Clínico Universitario de Valencia, Blasco Ibañez w/n, 46010 Valencia, Spain; 4 Nursing Department, Valencia University, Menédez i Pelayo n19, 46010 Valencia, Spain; 5 Nursing Wound Unit, Hospital Clínico Universitario de Valencia, Blasco Ibañez w/n, 46010 Valencia, Spain

**Keywords:** nurse, nursing, dependency-related skin injury, critical care units, skin injury, pressure injury, dermatitis

## Abstract

**Background/Objectives:** Dependency-related skin injuries (DRSIs) are a significant health issue that exacerbates in critically ill patients, as their clinical condition (immobility, hemodynamic instability, use of diagnostic and therapeutic devices, malnutrition, and pharmacotherapy) promotes their occurrence. The objective of this study was to analyze the incidence and characteristics of DRSIs in critically ill patients at a tertiary care hospital. **Methods:** An observational, descriptive, longitudinal, and prospective study was conducted in the Intensive Care Units of the Hospital Clínico Universitario de Valencia. Data were analyzed using R software (v.4.2.2). The study was approved by the institutional ethics committee, and informed consent was obtained from each patient or family member. **Results:** The final sample included 139 patients. The incidence of DRSIs was 33.1% (*n* = 46), with a total of 82 lesions recorded. The mean time to DRSI onset was 9.2 days. Pressure ulcers accounted for 76.8% (*n* = 63) of the injuries, of which 47.6% (*n* = 30) were category I. Approximately 40% of the DRSIs were associated with the use of medical devices, primarily endotracheal tubes, non-invasive ventilation, and respiratory support devices. The most frequent locations were the heels, sacrum, and nose. **Conclusions:** One-third of the patients admitted to the studied critical care units developed one or more DRSIs. The use of clinical devices and high-risk scores on risk assessment scales (EVARUCI and/or Braden) is associated with a greater predisposition to developing these skin injuries.

## 1. Introduction

Dependency-related skin injuries (DRSIs) represent a highly relevant health problem across all levels of care, particularly in critical care units. In these settings, patients’ clinical characteristics, together with the intensive use of diagnostic and therapeutic devices, significantly increase the risk of developing such lesions [1].

The National Advisory Group for the Study of Pressure Ulcers and Chronic Wounds (GNEAUPP) defines DRSI as “damage to the skin and/or underlying tissues affecting individuals with limitation or loss (temporary or permanent) of physical, mental, intellectual, or sensory autonomy due to disability, age, condition, or disease, who require assistance with basic activities” [2]. This concept includes pressure injury (PI), friction injuries (FIs), moisture-associated skin damage (MASD), and skin tears (STs), all of which are classified according to their etiology or causal mechanism [2,3]. The presence of DRSI is associated with poorer clinical outcomes, increased mortality, and a significant deterioration in patients’ quality of life [4].

Epidemiological data indicate that the burden of these injuries in critical care units continues to rise. In Spain, the most recent prevalence study conducted by the GNEAUPP (2022) reported an overall DRSI prevalence of 21.5% in Intensive Care Units (ICUs) and 14.8% in post-surgical and recovery units [5]. In both settings, PIs remained the most frequent type (16–18%), reflecting an increase compared to previous reports [6].

Regarding other etiologies, the national prevalence of MASD and incontinence-associated dermatitis (IAD) in critical care stands at 1.5% [5], though its incidence can reach up to 26.2% in patients under deep sedation [7]. Furthermore, friction and combined injuries show a prevalence of 0.9% [5] while medical adhesive-related skin injuries (MARSIs) present local incidence rates between 15% and 19%, commonly associated with the use of diagnostic and therapeutic devices [5].

On the international stage, large-scale research reflects similar challenges. The DecubICUs study—which analyzed more than 1100 ICUs across 90 countries—reported a prevalence of 16.2% for ICU-acquired pressure injuries [8]. For non-pressure etiologies, international studies report IAD rates ranging from 22.5% [9] to 26% [10]. MARSI exhibits a cumulative incidence of approximately 25.2% [11,12], whereas FIs range between 10% and 15% in post-surgical patients, increasing up to 44.9% in cases of prolonged prone positioning [13].

Numerous risk factors are involved in the development of DRSIs, many of which are modifiable through specific preventive interventions. Identifying these factors is essential for implementing effective strategies to reduce their occurrence. Consequently, longitudinal studies are required to better understand the holistic etiology of DRSIs [3] and to determine effective preventive measures by analyzing their onset and progression over time in critically ill patients.

To address this gap, this longitudinal descriptive study was conducted with the primary objective of determining the incidence of DRSIs in a population of critically ill patients. Secondary objectives include describing the characteristics of the observed lesions and identifying factors associated with their occurrence, as well as describing the preventive measures implemented in clinical practice.

## 2. Materials and Methods

### 2.1. Study Design and Setting

An observational, descriptive, longitudinal, and prospective study was conducted in accordance with the STROBE guidelines [14]. The aim was to analyze the incidence and characteristics of dependency-related skin injuries (DRSIs) in critically ill patients.

The study was carried out in the critical care units (Intensive Care Unit and Post-surgical Recovery Unit) of a tertiary-level hospital with a total capacity of 28 beds. Data collection took place from November 2023 to April 2024.

At the time of the study, no specific protocol for the prevention of DRSIs in critically ill patients had been implemented in the unit; however, the use of local preventive measures was standardized in the unit, including the application of sacral protective dressings, the use of Hyperoxigenated Fatty Acid (HOFA) and heel protectors, in accordance with the GNEAUPP recommendations.

### 2.2. Population and Selection Criteria

All patients aged over 18 years admitted to the participating units and with a length of stay longer than 48 h were included. Patients presenting any DRSI at admission or those who did not provide informed consent were excluded.

Patients remained in the study until death, hospital discharge, or transfer to another center.

### 2.3. Sample Size

The sample size of the present study was derived from the calculation performed for the parent project: a quasi-experimental pre–post study. Since this article constitutes the first phase of that research (baseline phase), the included sample guarantees the statistical power required for the subsequent full intervention.

Following a review of the available literature on incidence studies with similar characteristics to the present work, the mean incidence from two key studies was calculated as a baseline reference: Roca-Biosca et al. 2016 [15], which reported an incidence of 27%, and Valls-Matarín et al. 2021 [16], with an incidence of 45%. This yielded a pooled mean incidence of 36% as our starting point. Assuming a two-sided alpha risk of 0.05 and a statistical power greater than 0.80, a sample size of 122 subjects per group (122 in the pre-intervention group and 122 in the post-intervention group) is required to detect a statistically significant difference between two proportions. The expected proportions were set at 0.36 for Group 1 and 0.20 for Group 2, assuming a 0% follow-up loss rate.

### 2.4. Study Variables

#### 2.4.1. Independent Variables

Sociodemographic variables were collected, including age, sex, weight, height, and admission diagnosis. Clinical status variables were also recorded, such as incontinence, malnutrition, type of nutrition, laboratory values, presence of edema, use of vasoactive drugs, and hemodynamic instability.

Additionally, associated factors with the DRSIs development and the distribution of preventive measures were documented, including the use of diagnostic and/or therapeutic devices (nasogastric tube, restraints, urinary catheter, vascular catheters, oxygen therapy, mechanical ventilation, endotracheal tube or tracheostomy, continuous renal replacement therapy, drains, rectal collectors, and extracorporeal membrane oxygenation [ECMO]), patient positioning, use of pressure-relieving support surfaces, repositioning, skin hydration, and local pressure-relief devices.

Risk assessment was also performed at admission, during hospitalization, and at discharge using the Braden [17] and EVARUCI [18] scales.

#### 2.4.2. Dependent Variable

The primary outcome variable was the occurrence of DRSIs (yes/no).

For patients who developed lesions, data were collected on the type and number of lesions, as well as their characteristics, including category, location, causative factor, time of onset, association with clinical devices or pressure-relief surfaces, and whether resolution occurred before discharge (including time to healing when applicable).

### 2.5. Procedure and Data Collection

Beginning 24 h after admission, patients were assessed during hygiene care either daily or at a maximum interval of 48 h. Following clinical observation, variables were collected from the Electronic Health Record via the Orion Clinic system (Consellería de Sanitat, Valencian Community, Spain). To eliminate inter-observer variability, all data collection and clinical evaluations were performed by a single investigator, who had been formally trained and certified through the GNEAUPP SECLARED course.

Upon detection of a DRSI, specific lesion-related characteristics were documented. DRSIs were classified strictly according to the GNEAUPP guidelines, encompassing pressure injuries, friction injuries, moisture-associated skin damage, skin tears, and mixed lesions [2].

For subsequent statistical analysis, all anonymized data were compiled using Microsoft Excel (Office 365, Microsoft Corporation, Redmond, WA, USA).

### 2.6. Statistical Analysis

Descriptive data were presented as means and standard deviations, as well as medians and interquartile ranges for continuous quantitative variables. For qualitative variables, proportions and their 95% confidence intervals were calculated.

To identify risk and protective factors associated with the development of DRSIs, multiple univariate analyses were performed between the DRSI variable and the collected variables. Associations between qualitative variables were analyzed using Fisher’s exact test, while associations with quantitative variables were assessed using the Wilcoxon test.

Additionally, *p*-values were adjusted using the False Discovery Rate (FDR) method [19] to control for false positives in the context of multiple comparisons, ensuring the accuracy and reliability of the results.

A significance level of α = 0.05 was used for all analyses.

For the incidence model, the following variables were analyzed in relation to DRSIs using a regression model: length of stay (days), EVARUCI score, EVARUCI risk at discharge, Barthel Index, incontinence, nasogastric tube, mechanical ventilation, local pressure-relief devices, heel protectors, sacral dressings, and nutrition. The modeling approach followed the method proposed by Zuur and Ieno [20].

Initially, a saturated model including all variables (beyond optimal) was fitted using a binomial error distribution with a logit link function. The fixed-effects structure was then optimized through backward selection, comparing nested models using the Akaike Information Criterion (AIC). Model interpretation was performed through inspection of coefficients and ANOVA using the car package [21] (v.3.1-5). To facilitate clinical interpretability, odds ratios (ORs) and their 95% confidence intervals were calculated for each predictor retained in the final model.

All analyses were conducted using R software (v.4.2.2) [22].

### 2.7. Ethical Considerations

The study was approved by the Clinical Research Ethics Committee of the Hospital Clínico Universitario de Valencia.

Informed consent was obtained from all patients or their legal representatives—when patients were unable to provide consent autonomously—prior to inclusion in the study.

All data were collected and stored in strict compliance with current data protection regulations (Organic Law 3/2018 and Regulation [EU] 2016/679—GDPR). No direct or indirect identifying information was included that could lead to the identification of the patients participating in the study.

## 3. Results

### 3.1. Results Related to Demographic Variables

#### 3.1.1. Demographic Variables

The detailed results of the demographic variables and their impact on the occurrence of DRSI are presented in Table 1.

#### 3.1.2. Incidence

Of the 139 patients, 46 (33.1%) developed at least one DRSI. Among the specific types, pressure injuries (PIs) were the most frequent, affecting 34 patients (24.5% of the total). The mean time to onset of the first DRSI was 9.2 days, with skin tears appearing earliest (mean of 4.3 days) and mixed lesions appearing latest (mean of 12.8 days). The lesion burden was highest for PIs, with a mean of 1.65 lesions per affected patient. A detailed analysis is shown in Table 2.

### 3.2. Results Related to DRSI Incidence Associated Factors

The proportion of patients at maximum risk according to the EVARUCI scale was significantly higher in the group with DRSI (82.6%) compared to the group without lesions (34.4%). Likewise, a lower mean Barthel Index (15 vs. 30) and a higher frequency of advanced diagnostic and/or therapeutic support were observed in those who developed a DRSI.

Parallel to these clinical parameters, the recorded implementation of preventive measures was also more frequent in the DRSI group, with 95.7% of these patients receiving some form of local pressure relief device. A detailed analysis of all variables is presented in Table 3.

### 3.3. Incidence Model

The optimal model for DRSI incidence was determined through backward selection. Fitted to a binomial distribution, this model satisfies the necessary assumptions (homoscedasticity, absence of multicollinearity, and the absence of outliers).

As shown in Table 4, each additional day of hospital stay is associated with increasing the odds of developing a DRSI by 9%. A one-point increase in the EVARUCI score at admission was associated with a 10% increase in the odds of DRSI development.

The recorded use of local pressure-relief devices was also associated with the presence of DRSI.

In Section 3.4, in Table 5, we will see the association between perceived risk scales and the application of preventive measures.

### 3.4. Association Between Perceived Risk Scales and Implemented Preventive Measures

Statistical analysis revealed significant associations in nine of the studied variables based on the risk levels assessed by the EVARUCI and Braden scales (Table 5). The implementation of preventive measures recorded showed a heterogeneous distribution according to the patient’s risk profile.

Specifically, the application of preventive measures upon admission was significantly associated with scores indicating higher risk (higher EVARUCI scores and lower Braden scores). This significant association with higher risk levels was observed for the use of local pressure relief devices in general, and specifically for the employment of heel pads and sacral dressings.

Regarding nutritional therapy, enteral and parenteral nutrition were significantly associated with higher-risk profiles, whereas oral nutrition was linked to patients with lower risk on both scales.

In terms of repositioning, patients turned at intervals of less than or equal to 4 h presented significantly lower risk scores on both scales compared to those repositioned at intervals greater than 4 h. Conversely, the use of hyperoxygenated fatty acid (HOFA) showed a statistically significant association only with EVARUCI scores.

In contrast, the application of preventive measures after injury onset and the use of protective creams showed no statistically significant association with risk levels on either scale.

## 4. Discussion

The DRSI paradigm adapts to the context of critical care. The results of this study show a high incidence of DRSIs in critically ill patients (33.09%). PU was the predominant etiology, mostly in the early category, highlighting the relevant proportion of lesions associated with clinical devices. Overall, the presence of DRSI seems to be closely associated with longer hospital stays and higher risk scores on the EVARUCI scale. In general, preventive measures registered were more frequently implemented in higher-risk patients, although improvements are still needed in moisture management.

Compared to the existing literature, the observed incidence is higher than that reported in similar studies (27–28%) [15,23,24,25], which could be explained by the greater clinical complexity and severity of patients admitted to critical care units. As in previous studies, an association was identified between the occurrence of DRSIs and factors such as obesity, male sex, and prolonged hospital stay, reinforcing the idea that disease burden and dependency significantly influence the development of these lesions. The increased susceptibility of neurocritical patients is also consistent with previous findings [23], likely due to factors such as immobility, altered level of consciousness, and the use of sedation [26].

Regarding lesion characteristics, the predominance of pressure injuries (7 out of 10 lesions) is consistent with the available evidence [15,16,23], which identifies them as the main cutaneous adverse event in critical care. The mean time of onset (9–10 days) is consistent with the clinical course described in other studies, although the earlier onset of skin tears highlights skin vulnerability from the early stages of admission. The anatomical distribution observed, with frequent involvement of the heels, sacrum, buttocks, and facial areas, is also consistent with previously described patterns [23], particularly in relation to the use of clinical devices such as non-invasive mechanical ventilation and pulse oximetry sensors.

When analyzing each lesion type separately, pressure injuries showed a predominance of early categories (more than 50% category I), suggesting adequate early detection and the implementation of preventive measures. This finding is consistent with previous studies in which clinical surveillance can limit progression to more severe lesions [27].

The high proportion of injuries associated with clinical devices, especially invasive and non-invasive mechanical ventilation, confirms the role of these elements as possible contributors to their development, as widely reported in the literature [28,29,30,31,32]. In addition, our results also show the involvement of other devices, such as nasogastric tubes and urinary catheters, in line with previous studies [23].

Regarding MASD lesions, although their incidence was lower (7.19%) compared to other studies [33,34], they showed considerable severity at diagnosis, with category IIA predominating. In addition, their detection was later (around 9 days) compared to other studies reporting detection at 4–5 days after admission [7], suggesting possible deficits in early assessment. The strong association with incontinence (especially mixed, 73.91% in the DRSI group; *p* = 0.012) and their predominant location in the buttocks and perineal area are consistent with current evidence [7,33] and highlight the importance of moisture control in critically ill patients.

Friction injuries were mainly classified as category III and were associated with MARSI. Similarly, skin tears were entirely related to medical adhesives, although they showed a more balanced distribution across categories I, II, and III. These lesions were mainly located in the hip region, closely related to fixation systems used in compression bandages following invasive femoral procedures such as catheterization or embolization. Despite being preventable, their incidence and location vary widely in the literature and depend on patient-related factors such as age, comorbidities, and the type of adhesive used [35,36].

Mixed lesions (friction and pressure) were predominantly located on the heels. This may be related to psychomotor agitation or, conversely, to immobility requiring assisted mobilization, both of which increase shear forces.

Regarding factors associated with DRSIs incidence, the results confirm that higher EVARUCI scores (13.87 vs. 9.82; *p* < 0.001) and prolonged hospital stay are independent predictors of DRSIs, as shown in the multivariate model. When analyzing the differences in mean injury-free days between patients who developed DRSIs [7.13 ± 5.04] and those who did not [7.89 ± 5.3], the chronological relationship becomes complex. In clinical practice, determining whether a longer hospital stay leads to DRSI development or whether the occurrence of a DRSI extends hospitalization represents a classic epidemiological challenge. Rather than isolating these variables statistically, we understand that prolonged stay and injury development occur simultaneously, creating a bidirectional feedback loop where both factors reinforce each other.

Additionally, in the univariate analyses, several additional factors such as incontinence, use of nasogastric tubes (*p* = 0.036), endotracheal tubes (*p* = 0.044), mechanical ventilation (*p* = 0.028), and type of nutrition (*p* = 0.046) showed significant associations with DRSI occurrence, in line with previous models in critically ill patients [7]. However, in the multivariate model, these variables did not remain as independent predictors, likely due to their collinearity with the EVARUCI score and length of stay, which capture a broader dimension of clinical severity.

Regarding preventive measures recorded, greater implementation was observed in higher-risk patients, particularly pressure relief devices (present in 95.65% of patients with DRSIs), as well as heel pads and sacral dressings applied at admission in a high proportion of cases. This finding suggests early risk identification and targeted preventive strategies, consistent with current recommendations [37]. However, the significant association observed between these measures and the presence of DRSI must be interpreted with caution. Although a superficial analysis might falsely suggest that preventive interventions act as risk factors, this statistical association actually reflects a confounding by indication. Following good nursing practices, these preventive measures were intensively applied to patients presenting with greater clinical severity and baseline vulnerability. Therefore, rather than a direct risk factor, their high prevalence in the DRSI group serves as an indicator of the coexisting clinical risk, which heavily influences the statistical estimation of their protective effectiveness.

With regard to special pressure management surfaces (SPMSs), almost all patients were placed on high-technology active systems, which precludes comparison between different surface types. Nevertheless, their widespread use reflects the high complexity of critically ill patients and is supported by the literature as an effective strategy for pressure redistribution. Despite this, the incidence of DRSIs remains high, suggesting that these measures alone are not sufficient. This reinforces the multifactorial nature of DRSIs and the need for combined strategies including moisture control, nutritional optimization, and minimization of device-related adverse effects [15,23,24,25,30,31,32,33].

Following the nutritional optimization, in our study, enteral and parenteral nutrition were significantly associated with a higher risk of DRSIs according to the EVARUCI scale. This finding reflects the clinical severity and hypercatabolic state of critically ill patients, where the requirement for artificial nutritional support routinely coexists with malnutrition and hypoalbuminemia, both of which reduce tissue tolerance to pressure. Similarly, Serra et al. [38] demonstrated that serum albumin monitoring and administration decrease both the incidence and severity of skin injuries. Consequently, our results suggest that the need for artificial nutrition serves as an indirect marker of metabolic impairment, underscoring the importance of strict biochemical monitoring and intensified preventive measures in this patient profile.

Postural changes did not show a significant association with DRSI occurrence, which may be explained by clinical limitations in critically ill patients (e.g., hemodynamic instability, invasive devices, or deep sedation), reducing the feasibility and frequency of repositioning, as we observed in our results, with less frequency of postural changes in those patients at higher risk in the EVARUCI and BRADEN scales. However, current evidence continues to support their preventive role when applied with appropriate frequency and in combination with other measures [39].

Finally, moisture protection showed low implementation and was not systematically applied according to risk, particularly regarding the use of barrier creams. This is especially relevant given the higher severity observed in moisture-related injuries, indicating a clear area for improvement in clinical practice. Overall, although preventive measures are more frequently applied in high-risk patients, further optimization in their adequacy, individualization, and combination is needed to improve their effectiveness.

### 4.1. Limitations and Biases

These results should be interpreted in light of certain limitations. First, the limited number of longitudinal studies addressing the incidence of DRSI as a global entity makes direct comparison with the existing literature difficult.

Likewise, characteristics of the critical care environment, such as high workload, patient turnover, and the prioritization of life-support measures, may have influenced both data collection and the implementation of preventive interventions. Finally, the absence of a specific DRSI prevention protocol during the study period may have contributed to variability in the interventions applied and, consequently, in the results observed.

On the other hand, potential sources of bias should also be considered. First, selection bias may be present, as the sample was obtained from a single hospital and from critical care units with specific care characteristics, which may limit the generalizability of the findings to other settings.

Additionally, a methodological limitation of this study is the potential for time-at-risk bias and reverse causality regarding the length of hospital stay. Due to the observational nature of the data, it is challenging to completely isolate whether a prolonged stay acted strictly as an exposure factor for DRSI development, or whether the occurrence of the injury itself contributed to extending the hospitalization. Although this bidirectional relationship is a challenge in our research, length of stay is closely intertwined with clinical severity and other compounding patient factors. Consequently, for us, retaining this variable in the multivariable analysis is essential to accurately reflect the clinical dynamics of DRSI incidence in critically ill patients.

Finally, a possible confounding bias should be acknowledged, as patients with greater clinical severity—and therefore at higher risk of developing DRSI—were also those who most frequently received preventive measures (such as pressure relief devices or high-technology SPMS). This can be considered, on the one hand, as an indication bias from the perspective of research; however, in healthcare practice it is the correct clinical conduct, nursing should adapt preventive measures according to the risk of developing DRSI from admission as a mandatory quality standard, on the other hand, it could have hindered the ability to accurately assess the true association as a protective factor of these interventions.

### 4.2. Future Lines of Research

This study is part of a quasi-experimental research project, representing the preliminary (pre-intervention) phase. Following this incidence study, a training program on DRSI prevention in critical care was delivered to nursing professionals. Subsequently, a new phase of data collection and analysis will be conducted to evaluate post-training incidence and to assess the effectiveness of these educational interventions as a strategy for preventing DRSIs.

## 5. Conclusions

The findings of this study confirm that dependency-related skin injuries (DRSIs) represent a frequent and clinically relevant problem in critical care units, affecting approximately one third of patients. Pressure injuries were the most prevalent, many of them associated with the use of clinical devices.

The presence of DRSI is typically associated with a clinical profile characterized by high instability, elevated risk according to the EVARUCI scale, severe functional dependence, and exposure to invasive devices—particularly mechanical ventilation, endotracheal tubes, and nasogastric tubes—often in association with incontinence and advanced nutritional support. A prolonged hospital stay was also closely linked to the presence of these lesions, reflecting a complex clinical course.

Although preventive measures are intensively implemented in higher-risk patients, the higher rate of injuries in this subgroup underscores the clinical complexity and baseline vulnerability of these individuals, rather than a failure of the interventions themselves. These results highlight the need for early, individualized, and multimodal preventive strategies initiated immediately from the time of admission.

This manuscript was written in accordance with the STROBE statement for Cohort Studies [14]. 

## Figures and Tables

**Table 1:** Demographic variables and their relationship with the occurrence of DRSI.

Variable	No DRSI	Yes DRSI	Global	*p*-Value
N	93	46	139	
Age	63.47 ± 14.7	64.93 ± 13.11	63.96 ± 14.16	0.748
Sex [% (95% CI)]				
Female	48.39% (38.5–58.4%)	28.26% (17.32–42.55%)	41.73% (33.86–50.04%)	0.088
Male	51.61% (41.6–61.5%)	71.74% (57.45–82.68%)	58.27% (49.96–66.14%)	
Weight [x ± SD]	73.99 ± 15.11	78.7 ± 19.49	75.56 ± 16.77	
Height [x ± SD]	1.67 ± 0.09	1.69 ± 0.08	1.68 ± 0.08	
BMI [x ± SD]	26.52 ± 4.96	27.47 ± 6.05	26.83 ± 5.34	0.74
BMI Category [% (95% CI)]				
Underweight	1.16% (0.21–6.3%)	0% (6.94 × 10^−16^–8.2%)	0.78% (0.14–4.26%)	
Normal	46.51% (36.35–56.98%)	44.19% (30.43–58.89%)	45.74% (37.39–54.33%)	
Overweight	31.4% (22.56–41.82%)	25.58% (14.93–40.24%)	29.46% (22.28–37.83%)	
Obesity I	13.95% (8.17–22.82%)	18.6% (9.74–32.62%)	15.5% (10.27–22.74%)	
Obesity II	3.49% (1.19–9.76%)	2.33% (0.41–12.06%)	3.1% (1.21–7.7%)	
Obesity III	3.49% (1.19–9.76%)	9.3% (3.68–21.6%)	5.43% (2.65–10.78%)	
Origin [% (95% CI)]				
Emergency Department	62.37% (52.21–71.54%)	60.87% (46.46–73.61%)	61.87% (53.58–69.52%)	0.878
Inpatient Ward	7.53% (3.69–14.73%)	4.35% (1.2–14.53%)	6.47% (3.44–11.85%)	
Other Hospital	30.11% (21.73–40.07%)	34.78% (22.68–49.23%)	31.65% (24.5–39.79%)	
Unit [% (95% CI)]				
ICU	68.82% (58.81–77.33%)	60.87% (46.46–73.61%)	66.19% (57.98–73.52%)	0.624
Post-surgical ICU	31.18% (22.67–41.19%)	39.13% (26.39–53.54%)	33.81% (26.48–42.02%)	
Length of Admission (days)	7.89 ± 5.3	14.96 ± 10.62	10.23 ± 8.16	0.000056 *
[x ± SD]				
Injury-free days [x ± SD]	7.89 ± 5.3	7.13 ± 5.04	7.64 ± 5.3	0.291
Reason for Discharge [% (95% CI)]				
Death	13.98% (8.35–22.46%)	17.39% (9.09–30.72%)	15.11% (10.1–21.99%)	0.944
Discharge to Inpatient Ward	81.72% (72.66–88.26%)	78.26% (64.43–87.74%)	80.58% (73.21–86.29%)	
Another Hospital	4.3% (1.69–10.54%	4.35% (1.2–14.53%)	4.32% (1.99–9.1%)	
Diagnostic Group [% (95% CI)]				
Neurocritical	22.58% (15.27–32.07%)	32.61% (20.87–47.03%)	25.9% (19.33–33.76%)	0.733
Cardiovascular	31.18% (22.67–41.19%)	32.61% (20.87–47.03%)	31.65% (24.5–39.79%)	
Respiratory	19.35% (12.61–28.53%)	19.57% (10.65–33.17%)	19.42% (13.71–26.79%)	
Digestive	13.98% (8.35–22.46%)	6.52% (2.24–17.5%)	11.51% (7.21–17.88%)	
Other	12.9% (7.54–21.21%)	8.7% (3.43–20.32%)	11.51% (7.21–17.88%)	

DRSI: dependency-related skin injury; N: number of cases; CI: confidence interval; SD: standard deviation; *p*: statistical significance level; BMI: body mass index; and ICU: intensive care unit. Significant results are indicated by asterisks: *p* < 0.05: *; *p* < 0.1.

**Table 2:** DRSI incidence and lesion characteristics.

Variables	DRSI (*n* = 46)	PI (*n* = 34)	MASD (*n* = 10)	FI (*n* = 1)	Skin Tears (*n* = 5)	Mixed Lesions (*n* = 4)
N	82	56	11	1	8	6
Incidence [% (95% CI)]	33.09% (25.82–41.28%)	24.46% (18.06–32.23%)	7.19% (3.95–12.74%)	0.72% (0.13–3.96%)	3.6% (1.55–8.14%)	2.88% (1.12–7.17%)
Incidence rate (100 patients/day) [% (95% CI)]	4.33 [3.17–5.77]	3.20 [2.22–4.47]	0.94 [0.45–1.63]	0.09 [0.02–0.52]	0.47 [0.15–1.10]	0.37 [0.10–0.96]
Number of injuries [x ± SD]	0.64 ± 1.13	1.65 ± 1.12	1.11 ± 0.33	1	1.75 ± 0.96	1.5 ± 0.58
Days until appearance [x ± SD]	9.16 ± 6.78	9.62 ± 6.41	9.1 ± 6.85	11	4.25 ± 4.5	12.83 ± 10.23
Days for resolution [x ± SD]	0.17 ± 0.38	0.23 ± 0.43	0 ± 0	0	0.12 ± 0.35	0 ± 0
Days of cure [x ± SD]	11.21 ± 8.06	10.15 ± 7.3	-	-	25	-
Category [% (95% CI)]						
PI/FI Category I	36.59% (26.98–47.39%)	51.79% (39.01–64.33%)	-	0%	-	16.67% (3.01–56.35%)
PI/FI Category II	19.51% (12.38–29.37%)	21.43% (12.71–33.82%)	-	0%	-	66.67% (30–90.32%)
PI/FI Category III	18.29% (11.41–28.01%)	23.21% (14.1–35.77%)	-	100%	-	16.67% (3.01–56.35%)
PI Category IV	2.44% (0.67–8.46%)	3.57% (0.98–12.12%)	-	0%	-	0%
MASD Ia MASD Ib	2.44% (0.67–8.46%)	-	18.18% (5.14–47.7%)	-	-	-
MASD IIa	8.54% (4.2–16.59%)	-	63.64% (35.38–84.83%)	-	-	-
ST Category I	3.66% (1.25–10.21%)	-	-	-	37.5% (13.68–69.43%)	-
ST Category II	2.44% (0.67–8.46%)	-	-	-	25% (7.15–59.07%)	-
ST Category III	3.66% (1.25–10.21%)	-	-	-	37.5% (13.68–69.43%)	-
Lesion location [% (95% CI)]						
Heels	12.2% (6.76–21.01%)	7.14% (2.81–16.98%)	0%	0%	0%	100%
Occipital bone	4.88% (1.91–11.88%)	7.14% (2.81–16.98%)	0%	0%	0%	0%
Hip	4.88% (1.91–11.88%)	1.79% (0.32–9.45%)	0%	0%	37.5% (13.68–69.43%)	0%
Sacrum	13.41% (7.66–22.45%)	14.29% (7.42–25.74%)	27.27% (9.75–56.56%)	0%	0%	0%
Nose	12.2% (6.76–21.01%)	17.86% (10–29.84%)	0%	0%	0%	0%
Face	2.44% (0.67–8.46%)	3.57% (0.98–12.12%)	0%	0%	0%	0%
Ear	7.32% (3.4–15.06%)	10.71% (5–21.47%)	0%	0%	0%	0%
Foot	2.44% (0.67–8.46%)	3.57% (0.98–12.12%)	0%	0%	0%	0%
Neck	1.22% (0.22–6.59%)	1.79% (0.32–9.45%)	0%	0%	0%	0%
Back	3.66% (1.25–10.21%)	3.57% (0.98–12.12%)	0%	0%	12.5% (2.24–47.09%)	0%
Mouth	7.32% (3.4–15.06%)	10.71% (5–21.47%)	0%	0%	0%	0%
Legs	2.44% (0.67–8.46%)	1.79% (0.32–9.45%)	0%	0%	12.5% (2.24–47.09%)	0%
Arms	7.32% (3.4–15.06%)	8.93% (3.87–19.26%)	0%	0%	12.5% (2.24–47.09%)	0%
Buttocks	10.98% (5.88–19.56%)	3.57% (0.98–12.12%)	63.64% (35.38–84.83%)	0%	0%	0%
Hand	2.44% (0.67–8.46%)	3.57% (0.98–12.12%)	0%	0%	0%	0%
Perineal area	1.22% (0.22–6.59%)	0%	9.09% (1.62–37.74%)	0%	0%	0%
Other	3.66% (1.25–10.21%)	0%	0%	100%	25% (7.15–59.07%)	0%
Medical devices related [% (95% CI)]						
Global	40.24% (30.3–51.06%)	42.86% (30.77–55.86%)	0%	100%	100%	0%
Nasogastric tube	6.06% (1.68–19.61%)	8.33% (2.32–25.85%)	-	0%	0%	-
MARSI	24.24% (12.83–41.02%)	0%	-	100%	87.5% (52.91–97.76%)	-
Vascular access	12.12% (4.82–27.33%)	12.5% (4.34–31%)	-	0%	12.5% (2.24–47.09%)	-
NIMV interfaces	21.21% (10.68–37.75%)	29.17% (14.91–49.17%)	-	0%	0%	-
Endotracheal tube	36.36% (22.19–53.38%)	50% (31.43–68.57%)	-	0%	0%	-

DRSI: dependency-related skin injury; PI: pressure injury, MASD: moisture-associated skin damage; FI: friction injury; ST: skin tear; MARSI: medical adhesive-related skin injury; N: number of cases; CI: confidence interval; SD: standard deviation; *p*: statistical significance level; and NIMV: non-invasive mechanical ventilation.

**Table 3:** Descriptive analysis. Incidence of DRSI and associated factors.

Variable	No DRSI	Yes DRSI	Global	*p*-Value
N	93	46	139	
Cardiovascular Risk Factors [% (95% CI)]				
Yes	68.82% (58.81–77.33%)	60.87% (46.46–73.61%)	66.19% (57.98–73.52%)	0.624
No	31.18% (22.67–41.19%)	39.13% (26.39–53.54%)	33.81% (26.48–42.02%)	
Braden Risk on Admission [% (95% CI)]				
Without Risk	7.53% (3.69–14.73%)	4.35% (1.2–14.53%)	6.47% (3.44–11.85%)	0.147
Low Risk	23.66% (16.17–33.23%)	13.04% (6.12–25.67%)	20.14% (14.32–27.57%)	
Moderated Risk	33.33% (24.58–43.41%)	21.74% (12.26–35.57%)	29.5% (22.55–37.55%)	
High Risk	35.48% (26.51–45.61%)	60.87% (46.46–73.61%)	43.88% (35.91–52.19%)	
EVARUCI RISK [x ± SD]	9.82 ± 5.05	13.87 ± 3.73	11.16 ± 5.02	0.00014 *
Barthel Index [x ± SD]	29.98 ± 36.08	15.11 ± 26.4	25.06 ± 33.83	0.051
JH Downton [x ± SD]	1.72 ± 0.46	2.24 ± 0.56	1.97 ± 0.57	0.041 *
Stools [% (95% CI)]				
Liquid	8.6% (4.42–16.07%)	10.87% (4.73–23.04%)	9.35% (5.55–15.34%)	0.327
Pasty/soft	6.45% (2.99–13.37%)	13.04% (6.12–25.67%)	8.63% (5.01–14.48%)	
Melena	1.08% (0.19–5.84%)	4.35% (1.2–14.53%)	2.16% (0.74–6.15%)	
Hard	0% (0–3.97%)	2.17% (0.38–11.34%)	0.72% (0.13–3.96%)	
Normal	83.87% (75.08–89.97%)	69.57% (55.19–80.92%)	79.14% (71.64–85.06%)	
Incontinence [% (95% CI)]				
Continent	3.23% (1.1–9.06%)	0% (0–7.71%)	2.16% (0.74–6.15%)	0.012 *
Urinary incontinence	54.84% (44.73–64.56%)	26.09% (15.6–40.26%)	45.32% (37.28–53.61%)	
Fecal incontinence	1.08% (0.19–5.84%)	0% (0–7.71%)	0.72% (0.13–3.96%)	
Mixed incontinence	40.86% (31.43–51.02%)	73.91% (59.74–84.4%)	51.8% (43.56–59.94%)	
Diaper [% (95% CI)]	1.08% (0.19–5.84%)	0% (0–7.71%)	0.72% (0.13–3.96%)	1
Malnutrition [% (95% CI)]				
Normal nutritional status	55.91% (45.79–65.57%)	52.17% (38.14–65.88%)	54.68% (46.39–62.72%)	0.406
Risk of malnutrition	35.48% (26.51–45.61%)	45.65% (32.15–59.82%)	38.85% (31.15–47.15%)	
Malnutrition	8.6% (4.42–16.07%)	2.17% (0.38–11.34%)	6.47% (3.44–11.85%)	
Medical Devices [% (95% CI)]				
Nasogastric tube	44.09% (34.43–54.21%)	69.57% (55.19–80.92%)	52.52% (44.26–60.64%)	0.036 *
Mechanical restraint	21.51% (14.38–30.9%)	32.61% (20.87–47.03%)	25.18% (18.7–33%)	0.374
Bladder catheter	96.77% (90.94–98.9%)	100% (92.29–100%)	97.84% (93.85–99.26%)	0.729
Vascular access				
Peripheral catheter	24.73% (17.08–34.38%)	6.52% (2.24–17.5%)	18.71% (13.1–26%)	0.087
PICC line	10.75% (5.95–18.67%)	13.04% (6.12–25.67%)	11.51% (7.21–17.88%)	
CVC	64.52% (54.39–73.49%)	80.43% (66.83–89.35%)	69.78% (61.7–76.8%)	
Arterial catheter	87.1% (78.79–92.46%)	97.83% (88.66–99.62%)	90.65% (84.66–94.45%)	0.164
Oxygen therapy				
Nasal cannules	20.43% (13.49–29.72%)	10.87% (4.73–23.04%)	17.27% (11.89–24.41%)	0.299
Venturi mask	16.13% (10.03–24.92%)	13.04% (6.12–25.67%)	15.11% (10.1–21.99%)	
HFOT	27.96% (19.85–37.81%)	47.83% (34.12–61.86%)	34.53% (27.14–42.76%)	
Endotracheal tube	45.16% (35.44–55.27%)	50% (36.12–63.88%)	46.76% (38.67–55.03%)	0.044 *
Tracheostomy	3.23% (1.1–9.06%)	17.39% (9.09–30.72%)	7.91% (4.48–13.61%)	
Mechanical ventilation				
NIMV	13.98% (8.35–22.46%)	19.57% (10.65–33.17%)	15.83% (10.69–22.8%)	0.028 *
IMV	44.09% (34.43–54.21%)	65.22% (50.77–77.32%)	51.08% (42.85–59.25%)	
CRRT	7.53% (3.69–14.73%)	13.04% (6.12–25.67%)	9.35% (5.55–15.34%)	0.545
Drains	35.48% (26.51–45.61%)	43.48% (30.21–57.75%)	38.13% (30.48–46.42%)	0.624
Rectal collector	2.15% (0.59–7.51%)	13.04% (6.12–25.67%)	5.76% (2.94–10.95%)	0.056
Edema	33.33% (24.58–43.41%)	47.83% (34.12–61.86%)	38.13% (30.48–46.42%)	0.302
Hemodynamic instability	62.37% (52.21–71.54%)	73.91% (59.74–84.4%)	66.19% (57.98–73.52%)	0.356
Vasoactive drugs				
Norepinephrine	37.63% (28.46–47.79%)	52.17% (38.14–65.88%)	42.45% (34.54–50.76%)	0.374
Dobutamine	7.53% (3.69–14.73%)	8.7% (3.43–20.32%)	7.91% (4.48–13.61%)	
Prone position	4.3% (1.69–10.54%)	8.7% (3.43–20.32%)	5.76% (2.94–10.95%)	0.624
ECMO	2.15% (0.59–7.51%)	2.17% (0.38–11.34%)	2.16% (0.74–6.15%)	1
Preventive measures [% (95% CI)]				
Local pressure relief devices	64.52% (54.39–73.49%)	95.65% (85.47–98.8%)	74.82% (67–81.3%)	0.0004 *
Heel pads	45.16% (35.44–55.27%)	73.91% (59.74–84.4%)	54.68% (46.39–62.72%)	0.019 *
Sacral dressing	40.86% (31.43–51.02%)	67.39% (52.97–79.13%)	49.64% (41.45–57.85%)	0.028 *
Early sitting	34.41% (25.55–44.51%)	26.09% (15.6–40.26%)	31.65% (24.5–39.79%)	0.539
Postural changes				
No	1.08% (0.19–5.84%)	2.17% (0.38–11.34%)	1.44% (0.4–5.09%)	0.21
2 h	27.96% (19.85–37.81%)	17.39% (9.09–30.72%)	24.46% (18.06–32.23%)	
4 h	13.98% (8.35–22.46%)	2.17% (0.38–11.34%)	10.07% (6.09–16.2%)	
6 h	8.6% (4.42–16.07%)	6.52% (2.24–17.5%)	7.91% (4.48–13.61%)	
8 h	9.68% (5.18–17.38%)	13.04% (6.12–25.67%)	10.79% (6.65–17.04%)	
12 h	25.81% (18–35.53%)	45.65% (32.15–59.82%)	32.37% (25.16–40.54%)	
24 h	10.75% (5.95–18.67%)	13.04% (6.12–25.67%)	11.51% (7.21–17.88%)	
The patient’s condition does not allow it	2.15% (0.59–7.51%)	0% (0–7.71%)	1.44% (0.4–5.09%)	
Skin hydration				
HOFA	9.68% (5.18–17.38%)	21.74% (12.26–35.57%)	13.67% (8.93–20.36%)	0.273
Barrier cream	2.15% (0.59–7.51%)	0% (0–7.71%)	1.44% (0.4–5.09%)	
Moisturizer	88.17% (80.05–93.27%)	78.26% (64.43–87.74%)	84.89% (78.01–89.9%)	
Nutrition				
Absolute diet	11.83% (6.73–19.95%)	8.7% (3.43–20.32%)	10.79% (6.65–17.04%)	0.046 *
Oral nutrition	55.91% (45.79–65.57%)	30.43% (19.08–44.81%	47.48% (39.36–55.74%)	
Enteral nutrition	27.96% (19.85–37.81%)	50% (36.12–63.88%)	35.25% (27.8–43.49%)	
Parenteral nutrition	4.3% (1.69–10.54%)	10.87% (4.73–23.04%)	6.47% (3.44–11.85%)	
SPMS				
Dynamic SPMS	0% (0–3.97%)	2.17% (0.38–11.34%)	0.72% (0.13–3.96%)	0.539
Active high technology SPMS	100% (96.03–100%)	97.83% (88.66–99.62%)	99.28% (96.04–99.87%)	

CI: confidence interval; SD: standard deviation; *p*: statistical significance level; DRSI: dependency-related skin injury; PICC: peripherally inserted central catheter; CVC: central venous catheter; HFOT: high-flow oxygen therapy; NIMV: non-invasive mechanical ventilation; IMV: invasive mechanical ventilation; CRRT: continuous renal replacement therapy; ECMO: extracorporeal membrane oxygenation; HOFA: hyperoxygenated fatty acid; and SPMS: specialized pressure management surface. Significant results are indicated by asterisks: *p* < 0.05: *; *p* < 0.1.

**Table 4:** Incidence model. Type II ANOVA to evaluate the effect of each variable on the incidence of DRSI.

Variable	X^2^	DF	X^2^ *p*-Value	OR	CI 95% OR	OR *p*-Value
**Length of Admission (days)**	5.32	1	0.021 *	1.09	[1.03–1.16]	0.008 **
**EVARUCI Risk**	10.123	1	0.001 **	1.10	[1.00–1.21]	0.045 **
**Local Pressure Relief Devices**	5.705	1	0.017 *	5.66	[1.46–37.5]	0.028 *

X^2^: chi square; DF: degrees of freedom; *p*: statistical significance level; OR: odds ratio; CI: confidence interval; and DRSI: dependency-related skin injury. Significant results are indicated by asterisks: *p* < 0.01: **; *p* < 0.05: *; *p* < 0.1.

**Table 5:** Risk association and preventive measures recorded.

Variable	Comparison	EVARUCI Risk	*p*-Value	BRADEN Risk	*p*-Value
Local pressure relief devices					
At admission	No	8.15 ± 3.59	0.0002 ***	14.88 ± 3.88	0.000011 ***
	Yes	11.54 ± 4.91		11.68 ± 3.54	
During hospitalization	No	7.09 ± 2.94	0.0000013 ***	15.51 ± 3.83	0.0000014 ***
	Yes	11.63 ± 4.76		11.71 ± 3.49	
Heel pads					
Heel pads at admission	No	8.06 ± 3.53	0.00000003 ***	14.66 ± 3.74	0.000000013 ***
	Yes	12.75 ± 4.72		10.82 ± 3.12	
When the injury appears	No	11.19 ± 2.91	0.312	14.23 ± 4	0.004 **
	Yes	12.95 ± 4.61		10.45 ± 3.65	
During hospitalization	No	7.64 ± 3.14	1.5 × 10^−9^ **	14.9 ± 3.71	6.3 × 10^−9^ ***
	Yes	12.85 ± 4.66		10.82 ± 3.05	
Sacral dressing					
At admission	No	8.06 ± 3.53	0.00000003 ***	14.66 ± 3.74	0.000000013 ***
	Yes	12.75 ± 4.72		10.82 ± 3.12	
When the injury appears	No	12.07 ± 3.56	0.687	12.14 ± 4.1	0.27
	Yes	12.8 ± 4.86		11 ± 4.12	
During hospitalization	No	8.56 ± 3.7	0.0000071 ***	13.91 ± 4.11	0.00044 ***
	Yes	12.45 ± 5		11.41 ± 3.31	
Postural changes	>4 h	13.95 ± 4.87	5.2 × 10^−9^ ***	11 ± 3.25	0.0000012 ***
	≤4 h	7.11 ± 2.8		14.92 ± 3.61	
Barrier cream	No	10.5 ± 4.81	0.763	12.69 ± 3.93	0.621
	Yes	9.5 ± 4.95		11 ± 4.24	
HOFA	No	10.11 ± 4.64	0.024 *	12.72 ± 4	0.854
	Yes	12.87 ± 5.18		12.32 ± 3.53	
Nutrition	Absolut	10.27 ± 4.56	3.2 × 10^−11^ ***	11.2 ± 3.65	0.000000019 ***
	Oral	7.44 ± 2.69		14.76 ± 3.73	
	Enteral	14.39 ± 4.04		10.14 ± 2.07	
	Parenteral	12 ± 5.55		1.56 ± 4.88	

*p*: statistical significance level; HOFA: hyperoxygenated fatty acid. Significant results are indicated by asterisks: *p* < 0.001: ***; *p* < 0.01: **; *p* < 0.05: *; *p* < 0.1.

## Data Availability

The data presented in this study are available on request from the corresponding author due to findings of this study are currently not publicly available as they form part of an ongoing doctoral thesis.

## Public Involvement Statement

There was no public involvement in any aspect of this research.

## Guidelines and Standards Statement

This manuscript was drafted against the STROBE for Observational research.

## Abbreviations

The following abbreviations are used in this manuscript:

AIC	Akaike Information Criterion
BMI	Body Mass Index
CI	Confidence Interval
CRRT	Continuous Renal Replacement Therapy
CVC	Central Venous Catheter
DF	Degrees of Freedom
DRSI	Dependency-Related Skin Injury
ECMO	Extracorporeal Membrane Oxygenation
EMM	Estimated Marginal Means
FDR	False Discovery Rate
FI	Friction Injury
GNEAUPP	National Advisory Group for the Study of Pressure Ulcers and Chronic Wounds (Spanish translation)
HFOT	High-Flow Oxygen Therapy
HOFA	Hyperoxygenated Fatty Acid
IAD	Incontinence-Associated Dermatitis
ICU	Intensive Care Unit
IMV	Invasive Mechanical Ventilation
MARSI	Medical Adhesive-Related Skin Injury
MASD	Moisture-Associated Skin Damage
N	Number of Cases
NIMV	Non-Invasive Mechanical Ventilation
OR	Odds Ratio
*p*	Statistical Significance Level
PI	Pressure Injury
PU	Pressure Ulcer
PICC	Peripherally Inserted Central Catheter
SD	Standard Deviation
SPMS	Specialized Pressure Management Surface
ST	Skin Tear
STROBE	STrengthening the Reporting of OBservational studies in Epidemiology
USA	United States of America
X^2^	Chi Square

## References

[1] Soldevilla Agreda J.J., Torra Bou J.E., Verdú Soriano J., López Casanova P. (2011). 3rd National survey of the prevalence of pressure ulcers in Spain, 2009: Epidemiology and defining variables in lesions and patients. Gerokomos.

[2] García Fernández F.P., Soldevilla Ágreda J.J., Pancorbo Hidalgo P.L., Verdú Soriano J., López Casanova P., Rodríguez Palma M. (2021). Clasificación-categorización de las lesiones cutáneas relacionadas con la dependencia. Serie Documentos Técnicos GNEAUPP No II.

[3] García-Fernández F.P., Agreda J.J.S., Verdú J., Pancorbo-Hidalgo P.L. (2014). A new theoretical model for the development of pressure ulcers and other dependence-related lesions. J. Nurs. Scholarsh..

[4] José Avilés Martínez M., Alepuz Vidal L., Benítez Martínez J.C., Casaña Granell J., Clement Imbernón J., Fornes Pujalte B., Molina P.G., Tébar J.L.G., Casanova P.L., Mendoza M.M. (2012). Guía de Práctica Clínica Para el Cuidado de Personas con Úlceras por Presión o Riesgo de Padecerlas.

[5] García-Fernández F.P., Soldevilla-Agreda J.J., Pancorbo-Hidalgo P.L., Torra-Bou J.E., López-Franco M.D. (2023). Prevalencia de las lesiones cutáneas relacionadas con la dependencia en adultos hospitalizados en España: Resultados del 6.º Estudio Nacional del GNEAUPP 2022. Gerokomos.

[6] Pancorbo-Hidalgo P.L., García-Fernández F.P., Pérez-López C., Javier Soldevilla Agreda J. (2019). Prevalencia de lesiones por presión y otras lesiones cutáneas relacionadas con la dependencia en población adulta en hospitales españoles: Resultados del 5º Estudio Nacional de 2017. Gerokomos.

[7] Valls-Matarín J., Del Cotillo-Fuente M., Ribal-Prior R., Pujol-Vila M., Sandalinas-Mulero I. (2017). Incidencia de dermatitis asociada a la incontinencia en una unidad de cuidados intensivos. Enferm. Intensiv..

[8] Labeau S.O., Afonso E.P., Benbenishty J., Blackwood B., Boulanger C., Brett S.J., Calvino-Gunther S., Chaboyer W., Coyer F., Deschepper M. (2021). Prevalence, associated factors and outcomes of pressure injuries in adult intensive care unit patients: The DecubICUs study. Intensive Care Med..

[9] de Oliveira Ramalho A., de Oliveira Pacheco A., Aparecida Gonçalves Brandão A.C.M., Alves Silva R., de Souza Silva Freitas P. (2024). Prevalence of incontinence-associated dermatitis and associated factors in intensive care patients. ESTIMA Braz. J. Enteros. Ther..

[10] Kacmaz H.Y., Karadag A. (2023). Risk factors of incontinence-associated dermatitis among critically ill patients: A systematic review and meta-analysis. Front. Med..

[11] Zhang L., Chen J., Chen X., Yu P., Liu L., Chen M., Yuan X., Xu Y., Wang N., Zhu M. (2025). Medical Adhesive-related Skin Injury in Critically Ill Patients: A Systematic Review and Meta-analysis. J. Tissue Viability.

[12] Li Y., Chen X., Xie G., Xu L., He M., Fu W., Zhou Q. (2025). Incidence and Characteristics of Medical Adhesive-Related Skin Injuries in Patients Following Spinal Surgery: A Prospective Observational Study. Int. J. Nurs. Stud. Adv..

[13] Chaboyer W., Thalib L., Harbeck E.L., Coyer F.M., Blot S., Bull C.F., Nogueira P.C., Lin F.F. (2022). Incidence and Prevalence of Pressure Injuries in Adult Intensive Care Patients: A Systematic Review and Meta-Analysis. J. Wound Ostomy Cont. Nurs..

[14] Von Elm E., Altman D.G., Egger M., Pocock S.J., Gøtzsche P.C., Vandenbroucke J.P., STROBE Initiative (2007). The Strengthening the Reporting of Observational Studies in Epidemiology (STROBE) statement: Guidelines for reporting observational studies. Ann. Intern. Med..

[15] Roca-Biosca A., Rubio-Rico L., de Molina-Fernández M.I., Tuset-Garijo G., Colodrero-Díaz E., García-Fernández F.P. (2016). Incidencia de lesiones relacionadas con la dependencia en una población de pacientes críticos. Enferm. Clínica.

[16] Valls-Matarín J., Peradejordi-Torres R.M., Del Cotillo-Fuente M. (2023). Dependency-related skin lesions in the prone critical patient: Incidence study. Enferm. Clin..

[17] Bergstrom N., Braden B.J., Laguzza A., Holman V. (1987). The Braden Scale for Predicting Pressure Sore Risk. Nurs. Res..

[18] González-Ruiz J.M., Núñez-Méndez P., Balugo-Huertas S., Navarro-de la Peña L., García-Martín M.R. (2008). Estudio de validez de la Escala de Valoración Actual del Riesgo de desarrollar Úlceras por presión en Cuidados Intensivos (EVARUCI). Enferm. Intensiv..

[19] Benjamini Y., Hochberg Y. (1995). Controlling the false discovery rate: A practical and powerful approach to multiple testing. J. R. Stat. Soc. Ser. B (Methodol.).

[20] Zuur A.F., Ieno E.N. (2016). A protocol for conducting and presenting results of regression-type analyses. Methods Ecol. Evol..

[21] Fox J., Weisberg S. (2019). An R Companion to Applied Regression.

[22] R Core Team (2022). R: A Language and Environment for Statistical Computing.

[23] Roca-Biosca A., García-Fernández F.P., Chacón-Garcés S., Rubio-Rico L., de Molina-Fernández M.I., Anguera-Saperas L., García-Grau N., Tuset-Garijo G., Guillén M.d.C.V., Colodrero-Díaz E. (2015). Identificación y clasificación de las lesiones relacionadas con la dependencia: De la teoría a la práctica clínica. Gerokomos.

[24] Monteiro D.S., Borges E.L., Spira J.A.O., Garcia T.d.F., de Matos S.S. (2021). Incidence of skin injuries, risk and clinical characteristics of critical patients. Texto Contexto-Enferm..

[25] Becker D., Tozo T.C., Batista S.S., Mattos A.L., Silva M.C.B., Rigon S., Laynes R.L., Salomão E.C., Hubner K.D.G., Sorbara S.G.B. (2017). Pressure ulcers in ICU patients: Incidence and clinical and epidemiological features: A multicenter study in southern Brazil. Intensive Crit. Care Nurs..

[26] Cox J. (2011). Predictors of pressure ulcers in adult critical care patients. Am. J. Crit. Care.

[27] Rodríguez-Núñez C., Iglesias-Rodríguez A., Irigoien-Aguirre J., García-Corres M., Martín-Martínez M., Garrido-García R. (2019). Registros enfermeros, medidas de prevención e incidencia de úlceras por presión en una Unidad de Cuidados Intensivos. Enferm. Intensiv..

[28] Health Research & Educational Trust (2017). Acquired Pressure Ulcers/Injuries (HAPU/I).

[29] Black J.M., Cuddigan J.E., Walko M.A., Didier L.A., Lander M.J., Kelpe M.R. (2010). Medical device related pressure ulcers in hospitalized patients. Int. Wound J..

[30] Gregoretti C., Confalonieri M., Navalesi P., Squadrone V., Frigerio P., Beltrame F., Carbone G., Conti G., Gamna F., Nava S. (2002). Evaluation of patient skin breakdown and comfort with a new face mask for noninvasive ventilation: A multi-center study. Intensive Care Med..

[31] Carron M., Freo U., BaHammam A.S., Dellweg D., Guarracino F., Cosentini R., Feltracco P., Vianello A., Ori C., Esquinas A. (2013). Complications of non-invasive ventilation techniques: A comprehensive qualitative review of randomized trials. Br. J. Anaesth..

[32] Moser C.H., Peeler A., Long R., Schoneboom B., Budhathoki C., Pelosi P.P., Brenner M.J., Pandian V. (2022). Prevention of Endotracheal Tube-Related Pressure Injury: A Systematic Review and Metaanalysis. Am. J. Crit. Care.

[33] Bliss D.Z., Savik K., Thorson M.A.L., Ehman S.J., Lebak K., Beilman G. (2011). Incontinence-Associated Dermatitis in Critically Ill Adults: Time to Development, Severity, and Risk Factors. J. Wound Ostomy Cont. Nurs..

[34] Wang X., Zhang Y., Zhang X., Zhao X., Xian H. (2018). Incidence and risk factors of incontinence-associated dermatitis among patients in the intensive care unit. J. Clin. Nurs..

[35] Frota O.P., Pinho J.N., Ferreira-Júnior M.A., Sarti E.C.F.B., Paula F.M., Ferreira D.N. (2023). Incidence and risk factors for medical adhesive related skin injury in catheters of critically ill patients: A prospective cohort study. Aust. Crit. Care.

[36] Fumarola S., Allaway R., Callaghan R., Collier M., Downie F., Geraghty J., Kiernan S., Spratt F., Bianchi J., Bethell E. (2020). Overlooked and underestimated medical adhesive-related skin injuries. J. Wound Care.

[37] Bambi A.A.S., Yusuf S.S., Irwan A.M.S. (2022). Reducing the Incidence and Prevalence of Pressure Injury in Adult ICU Patients with Support Surface Use: A Systematic Review. Adv. Skin. Wound Care.

[38] Serra R., Grande R., Buffone G., Gallelli L., Caroleo S., Tropea F., Amantea B., de Franciscis S. (2015). Albumin administration prevents the onset of pressure ulcers in intensive care unit patients. Int. Wound J..

[39] Barahona-Vimos J.P., Rodríguez-Plascencia A., Romero-Fernández A.J. (2025). Beneficios de los cambios posturales para la prevención de lesiones por presión en pacientes críticos. Salud Vida.

